# Drowning in emails: investigating email classes and work stressors as antecedents of high email load and implications for well-being

**DOI:** 10.3389/fpsyg.2024.1439070

**Published:** 2024-10-03

**Authors:** Marcel Kern, Sandra Ohly, Lenka Ďuranová, Juliane Friedrichs

**Affiliations:** ^1^Faculty of Psychology, Ruhr University Bochum, Bochum, Germany; ^2^Department of Business Psychology, University of Kassel, Kassel, Germany; ^3^Faculty of Business and Economics, University of Applied Sciences Schmalkalden, Schmalkalden, Germany; ^4^Faculty of Business and Law, Aschaffenburg University of Applied Sciences, Aschaffenburg, Germany

**Keywords:** email, email demands, email load, action regulation theory, cross-lagged panel, well-being

## Abstract

**Introduction:**

High email load has been associated with impaired well-being because emails impose specific demands, disturb the workflow, and thereby overtax individuals’ action regulation toward prioritized goals. However, the causes and well-being-related consequences of email load are not yet well understood, as previous studies have neglected the interaction type and function of emails as well as co-occurring stressors as antecedents of high email load and have relied predominantly on cross-sectional designs.

**Methods:**

In two studies, we aimed to clarify the nature of email load through the lens of action regulation theory. The first study, a two-wave investigation with a fortnightly interval, examined the lagged relationships among email load, work stressors, strain, and affective well-being. The sample included 444 individuals across various occupations and organizations, with 196 of them working from home or remotely at least part of the time. In the second cross-sectional study, we surveyed 257 individuals using a convenience sampling approach, 108 of whom worked from home or remotely at least partially. This study focused on evaluating how different email classes—distinguished by email interaction type (received vs. processed) and email function (communication vs. task)—serve as predictors of high email load.

**Results:**

In Study 1, we found a positive lagged effect of high email load on strain, even when controlling for the co-occurring stressors time pressure and work interruptions. In addition, lagged effects of email load on time pressure and interruptions were identified, while no evidence was found for the reverse direction. The results of Study 2 suggest that only the number of communication-related emails received, but not the number of task-related emails received, or the number of all emails processed contribute to high email load.

**Conclusion:**

Findings suggest that email load can be considered a unique stressor and that different classes of email need to be distinguished to understand its nature. Clarifying the sources of email load can help develop effective strategies to address it.

## 1 Introduction

Despite the fact that a variety of communication channels have evolved over the past two decades, email is still the primary form of communication in business (Rosen et al., 2019; Steffensen et al., 2022). A survey of full-time employees in the United States and the United Kingdom revealed that email remains the primary communication tool (Axios, 2022) and is expected to continue in this role (The Radicati Group, 2023). Employees, and in particular knowledge workers, typically spend a large part of their daily working time reading and responding to emails (Adobe, 2019; Barley et al., 2011). The high prevalence of email is mainly due to its advantages, which include increased opportunities for knowledge sharing (Yuan et al., 2013), rapid international communication (Gibson and Gibbs, 2006), time flexibility (Boswell and Olson-Buchanan, 2007), and asynchrony (Byron, 2008).

Previous research has shown that these benefits may come with a whole host of undesirable consequences. For example, it has been found that emails are positively associated with interruptions (Jackson et al., 2003), additional work (Barley et al., 2011), and negatively with psychological detachment (Tedone, 2022) or goal progress (Rosen et al., 2019). Individuals faced with a high volume and low quality of emails are more likely to experience burnout and compromised well-being (e.g., Brown et al., 2014; Reinke and Chamorro-Premuzic, 2014). Evidence from non-academic sources suggests that people typically devote approximately 30–40% of their workweek to reading and responding to emails (Adobe, 2019; Atkin, 2012), many of which are irrelevant to their immediate tasks (Addas and Pinsonneault, 2018). A survey by Edison Mail (2022) found that 66% of Americans reported feeling stressed due to the volume of email messages they receive, adding to the growing concern. This phenomenon of being inundated with a too many and low-quality emails is often referred to as high email load and has garnered significant attention in research (McMurtry, 2014; Soucek and Moser, 2010; Sumecki et al., 2011).

Notwithstanding these findings, we lack a clear theoretical framework to predict when work-email activity is beneficial and when it leads solely to strain (Russell et al., 2024). Previous research has largely relied on qualitative data (Bellotti et al., 2005) or cross-sectional designs (e.g., Brown et al., 2014; Reinecke et al., 2017), and is thus limited in two ways. First, such findings do not allow to determine the direction of effects, so it is not yet clear whether email load precedes strain and impaired well-being or is more likely the result of it. This knowledge is essential, as it guides the development of effective strategies to support employees’ well-being and productivity. It will help determine whether interventions should focus on organizational-level measures, such as implementing policies to regulate email use, or on individual-level support, such as training employees to manage stress. Without this clarity, efforts to mitigate the negative impact attributed to email load may be misdirected. Second, employees were found to blame their email for the stress they experience at work, whether or not it is related to email, as the sheer number of emails in their inbox reminds employees of the work that still needs to be done (Barley et al., 2011). There is reason to believe that email load is strongly tied to other stressors related to work, requiring a consideration of the broader work environment. Specifically, individuals may be experiencing high email load only because they are exposed to high workloads, to performance constraints that impede work efficiency, to interruptions by others at work that keep employees from working, or to other stressors independent of email demands (Barley et al., 2011). This would imply that negative associations between email load and well-being can be explained, at least in part, by the presence of these stressors. Identification of the unique contribution of email load to the prediction of strain and well-being, independent of co-occurring stressors, is therefore important to our understanding of how work should be designed or improved to mitigate negative effects on employees.

**Figure 1:**
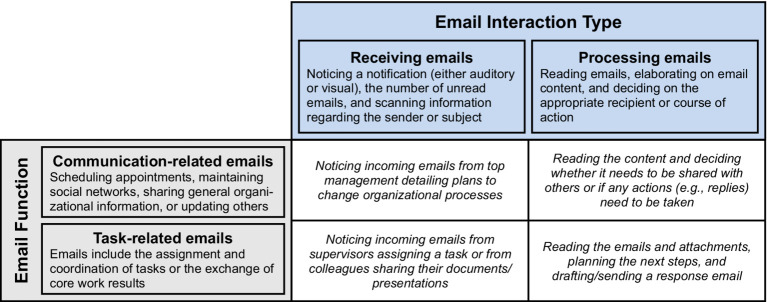
The distinction between email interaction type and email function and examples for each email class.

Another shortcoming of previous research is that studies have attributed email load mainly to the high volume and low quality of emails (Brown et al., 2014; Gupta et al., 2011; Sobotta, 2016; Stich et al., 2019a, 2019b). However, hardly any study has yet investigated whether this holds true for different classes of emails. Building on Wang et al.'s (2020) differentiation of information and communication technology (ICT) use by intensity and function, we distinguish between receiving emails and processing emails as different email interaction types, and between the function of emails, resulting in the identification of four distinct email classes: receiving communication-related emails, receiving task-related emails, processing communication-related emails, and processing task-related emails. Drawing upon the classification of work stressors proposed in action regulation theory—namely, regulation obstacles (i.e., stressors that directly impede goal attainment), regulation uncertainties (i.e., unclear goals and plans), and overtaxing regulations (i.e., quantitative stressors demanding increased effort or speed; Frese and Zapf, 1994; Hacker, 2003)—we contend that these four email classes differentially influences when employees actually experience high load (see Figure 1). Given this rationale, it seems imperative to consider these specific antecedents of email load.

**Figure 2:**
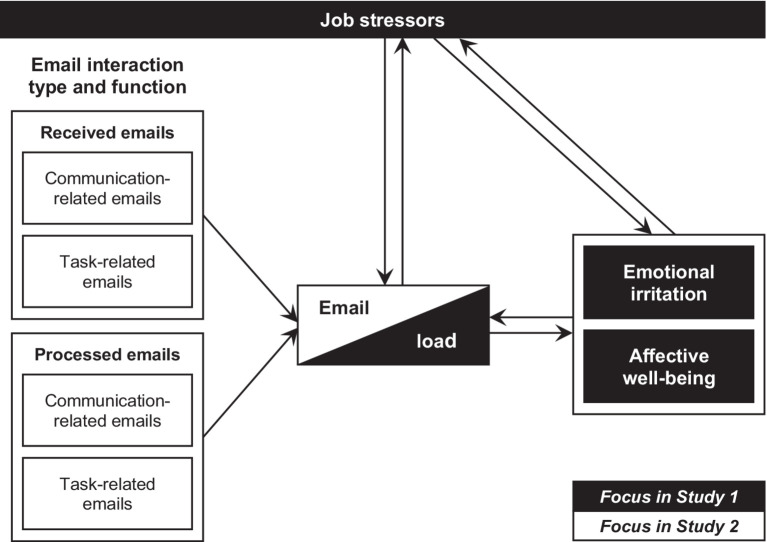
Conceptual research model.

Addressing these research gaps contributes to the literature in several ways: By examining the interplay of email load and other job stressors, and by investigating a differentiation of emails classes as antecedents, we aim to answer the pressing question raised in Hu et al.’s (2021) review of “how much each new construct contributes to the literature beyond what we have been studying in a more general context” (p. 386). In other words, how does email load contribute to strain and well-being beyond what is already known from previous research on work-related stress? We rely on two studies to examine if and why email load is harmful (see conceptual research model in Figure 2).

First, we test in a two-wave study the competing hypotheses of whether high email load is a unique stressor that increases strain and impairs well-being over time, or whether it results from employees being strained. The latter describes reverse causation that must be considered as equally likely (Guthier et al., 2020), as individuals who are highly stressed may not have the energy or the strategies to successfully process incoming information, such as deciding which email is important and which is not (see de Lange et al., 2004). By clarifying whether email load is truly the cause of decreased well-being or rather a byproduct of other factors such as high stress levels, we contribute to a better and unequivocal integration of email load into occupational stress models and facilitate the development of targeted and effective interventions.

Second, in the cross-sectional Study 2, we examine which specific emails classes (categorized by email interaction type and function) are most responsible for high email load (defined as excessive email demands interfering with action regulation), thereby challenging the commonly held belief that email volume alone is generally detrimental. Based on action regulation theory (Frese and Zapf, 1994; Hacker, 2003), we propose that processing a large volume of emails is functional for goal achievement, reflecting effective goal-related action regulation. In contrast, notifying a high volume of less relevant incoming emails disrupts action regulation toward salient goals. Complementing the distinction between *received and processed emails*, we propose to consider the function of email in relation to email load. Because working on tasks refers to primary tasks, and communicating with others largely to secondary tasks (Gupta et al., 2011), we expect differential effects for *task-related and communication-related emails.* By differentiating between these four email classes, we add to research that has employed similar distinctions within the broader context of ICT (e.g., Dietz et al., 2022), emphasizing that the impact of ICT and email use can only be fully understood by considering their relevance to one’s current work tasks.

## 2 High email load conceptualized in action regulation theory

Action regulation theory focuses on the cognitive regulation of action to elucidate how people pursue goals within the work context (Frese and Zapf, 1994; Hacker, 2003). We focus on its taxonomy of stressors, which classifies work stressors in terms of how they disturb action regulation toward goals (Frese and Zapf, 1994), and ultimately affect well-being. Three major types of so-called regulation problems can be distinguished: first, stressors can be *regulation obstacles* that refer to task-related events or conditions and make it harder or even impossible to achieve a certain goal. Typical examples are work interruptions, computer failures or poor work organization (Irmer et al., 2019). Second, stressors exist in the form of *regulation uncertainties*, meaning that a person does not know how to achieve a particular work goal. The well-known concepts of role conflict and role ambiguity belong to this category (Zacher and Frese, 2018). And third, stressors that demand a high speed or intensity of action regulation to achieve a goal are categorized as *overtaxing regulation*. Typically, this category includes stressors such as time pressure and concentration demands (Frese and Zapf, 1994; Zacher and Frese, 2018).

Following action regulation theory, high email load may be understood as an overtaxing regulation because high speed and intensity of action regulation are required to deal with email volume. This notion matches with Frese and Zapf’s (1994) classification of information load as an overtaxing regulation. Email load can be considered a special form of information load, but with a focus on electronic communication (Piecha and Hacker, 2020; Soucek and Moser, 2010). However, email load may also be classified as a regulation uncertainty in cases when incoming emails lack pertinent information and fail to align with salient work objectives (Burgess et al., 2005; Soucek and Moser, 2010), leading to ambiguity regarding their implications for one’s work and how to effectively manage them. Here, the lack of clarity and relevance, which are core characteristics of regulation uncertainties (Frese and Zapf, 1994), should be responsible for strain responses. Finally, email load may also be classified as a regulation obstacle to the extent that employees get interrupted by incoming email (Addas and Pinsonneault, 2018), need to obtain additional information from the email sender, decide which information must be heeded and which must not, and select which content is relevant and calls for action and which can be ignored, all of which making one’s work harder than necessary. Frese and Zapf (1994) refer to this type of regulation problem as the “informational difficulties” of the action process (p. 311). This classification of email load aligns with previous theoretical considerations emphasizing the quality of work-related emails (Dabbish and Kraut, 2006; Whittaker and Sidner, 1996).

Regardless of the specific classification, it can be inferred that high email load may be a regulatory stressor associated with detrimental consequences for one’s well-being, as has been well documented for all types of regulation problems in general (Irmer et al., 2019) and for high email load in particular (e.g., Misra and Stokols, 2012; Reinke and Chamorro-Premuzic, 2014). For this reason, we expect that high email load is associated with strain and impaired well-being over time.

*Hypothesis 1*: High email load has a positive lagged effect on psychological strain (a) and a negative lagged effect on well-being (b).

### 2.1 High email load as a consequence of strain and impaired well-being

According to the stressor perception hypothesis (de Lange et al., 2004; Guthier et al., 2020), strained employees may experience higher levels of work stressors, although the objective levels of stressors may not have changed. The rationale behind this proposition is that employees tend to evaluate their working conditions to be worse when they feel they do not have sufficient energy and resources to successfully complete their tasks. Strained employees are therefore more likely to report high email load when they feel unable to adequately handle the volume or complexity of incoming information in their emails. Evidence for this effect comes from a recent meta-analysis by Guthier et al. (2020) showing that the reverse effects of strain on stressors “were considerably larger” than the commonly assumed effects of stressors on strain (p. 1161). This may also apply to the lagged relationships between email load, strain, and well-being. Based on this theorizing and the related empirical results, we posit:

*Hypothesis 2*: Strain has a positive (a) and well-being a negative (b) lagged effect on email load.

### 2.2 High email load as a consequence of other work stressors

High email load can also be the result of work stressors because emails often remind a person of the work to be done (Barley et al., 2011) and because unpredictable email interruptions require cognitive suppression of impulses and emotion regulation (Rosen et al., 2019). Thus, it is not surprising that high email load has already been linked to time pressure and work interruptions. MacDonald et al. (2011), for instance, observed in their qualitative study that individuals felt stressed primarily due to time constraints, rather than an overwhelming amount of information. Such findings highlight the need to investigate email load along with the broader work context, which is why the present study considers time pressure and work interruptions as predictors of high email load.

Time pressure refers to the discrepancy between the amount of work to be done and the time available, or the requirement to work at high speed (Ohly and Fritz, 2010). When employees feel pressed for time, they are more likely to perceive that they are unable to handle their emails, leading to high email load. The rationale behind this is that time pressure can be considered as a strong situation (Mischel, 1977; see also Kern and Zapf, 2021) that requires employees to mobilize resources in order to maintain the attainment of high priority goals (Meijman and Mulder, 1998). These resources are then not available for dealing with other demands, such as reading and replying to emails, which is why emails are more likely to exceed one’s information processing capacity. This may explain why time pressure increases email load over time. Empirical evidence comes from research on information load, showing that it was rated higher when individuals were under high time pressure (Hahn et al., 1992; Kock, 2000).

Work interruptions refer to “temporary suspensions of goal-directed action” (Baethge and Rigotti, 2013, p. 43) and create a need to invest time and effort in mastering the interrupting task or issue before being able to fully return to a task (Leroy, 2009). Employees can be interrupted by a variety of sources, including supervisors, colleagues, and customers (for an overview, see Puranik et al., 2020). Time and effort are bound for dealing with interruptions and are, in result, no longer available for reading and replying to emails, which is why email load likely increases, especially if interruptions by others occur frequently. For these reasons, we posit that high email load is predicted by work interruptions:

*Hypothesis 3*: Time pressure (a) and work interruptions (b) have a positive lagged effect on email load.

### 2.3 High email load predicting work stressors

In line with the aforementioned reverse effects, we also explore reverse effects of email load on the work stressors time pressure and work interruptions, as they appear equally plausible. According to the stressor creation hypothesis (Spector et al., 2000; Zapf et al., 1996), overloaded employees may themselves create more stress in their work. When employees feel overwhelmed by the emails they need to process, they may use inefficient strategies to manage it, such as switching back and forth between processing emails and performing other tasks. This is time and energy consuming, leads to an accumulation of unfinished tasks, and can therefore increase time pressure over time (see Barley et al., 2011; Fenner and Renn, 2010). In addition, scholars have emphasized that emails directly generate supplementary tasks (e.g., filing and sorting emails or documents) that have to be addressed within working hours, thereby amplifying time pressure (for an overview, see Barley et al., 2011).

High email load will also lead to employees interrupting their work more frequently. High email traffic has been associated with loss of concentration (Mark et al., 2016), mistakes (Bailey and Konstan, 2006), as well as more inefficient communication (Soucek and Moser, 2010), thereby increasing workflow disruptions. When dealing with a high volume of emails, it is increasingly likely that they will be processed incorrectly or incompletely, for example by responding only partly to the request (Bailey and Konstan, 2006), by creating additional messages, or by people interrupting in person to clarify issues and provide additional information (see also Jett and George, 2003). Such considerations suggest that reverse lagged effects should also be considered, which is why we expect:

*Hypothesis 4*: Email load has a positive lagged effect on time pressure (a) and work interruptions (b).

## 3 Email-specific antecedents of high email load

### 3.1 The distinction between received and processed emails

Most previous studies have focused on email volume (also labeled email quantity) as the main antecedents of high email load (Brown et al., 2014; Dabbish and Kraut, 2006). With reference to action regulation theory (Frese and Zapf, 1994; Hacker, 2003), we believe that this view falls short, as different aspects of email use affects employees’ action regulation differently (for a review, see Russell et al., 2024). For this reason, we propose to distinguish between the number of received and processed emails to better understand when employees experience high email load. Interestingly, whereas previous studies have recorded both the number of emails received and the number of emails processed (e.g., Brown et al., 2014), they have usually combined them into one scale and analyzed them as a higher-level construct. However, receiving and processing emails constitute different demands in terms of action regulation (see Figure 1).

Incoming emails often arrive at unpredictable times and can interrupt workflow (Gupta et al., 2011), especially when signals about incoming mails are activated. Such “intrusions” (Jett and George, 2003) disrupt the continuity of cognitive processing of a task being pursued at that moment and require adjustment of action programs and plans to continue the work (Russell et al., 2007, 2024). With an increasing number of emails, it becomes more and more difficult to complete primary work tasks (Rosen et al., 2019; Russell et al., 2022). In this case, high email load represents an regulation obstacle according to action regulation theory (Frese and Zapf, 1994). When scanning emails for sender and subject, the lack of clarity regarding their implications for current work tasks (Burgess et al., 2005) may also lead to regulation uncertainty and thus hinder efficient action regulation.

Processing emails, in contrast, is either the result of employees trying to cope with incoming emails or represent self-initiated work behavior toward goals. In light of action regulation theory (Frese and Zapf, 1994; Hacker, 2003), processing emails can be considered as an attempt to restore normal action regulation by actively addressing the cause of high load or by coping with work demands. Nevertheless, processed emails may also be responsible for email load because they can still overtax action regulation: The processing of emails is associated with regulatory effort, which consumes energy and resources (Edwards, 1988). Complex cognitive processes are required to read and write emails, e.g., to understand the content, deal with the information included, and compose a structured message (Russell et al., 2007; Whittaker and Sidner, 1996). However, the regulatory effort involved in processing emails is often routinized (Ohly et al., 2017) and can also yield benefits such as goal accomplishment (empty inbox or finishing a work task), which may counteract the negative effects. Consequently, the experienced load resulting from received emails is likely to emanate from disturbed action regulation (i.e., regulation obstacles and regulation uncertainty) and processed emails may be linked to strain and impaired well-being because of the regulatory costs (i.e., overtaxing regulation). In addition, email function needs to be considered, which we turn to next.

### 3.2 The role of email function for email load

The extent to which emails are responsible for high email load is likely to vary depending on their function (Rice and Leonardi, 2014; Wang et al., 2020). Drawing from action regulation theory, communication-related emails (e.g., newsletters, general information on regulations, social events, scheduling appointments) are more likely to contribute to high email load compared to task-related emails because of their increased likelihood of containing ambiguous information. Even if not (fully) read, communication-related emails can cause frustration (Sumecki et al., 2011) by conflicting with important goals and diverting attention from what needs to be accomplished (Addas and Pinsonneault, 2018), particularly when notifications for incoming emails are enabled (Jett and George, 2003). They are often sent to a larger group of recipients to keep others informed of general news, even if a receiving employee is not directly involved in the matter (Gupta et al., 2006; Stich et al., 2019b). In many cases, the information transmitted is not relevant for immediate action toward prioritized goals, but must be stored in memory until it is needed (Piecha and Hacker, 2020). As a consequence, individuals who receive communication-related emails must first find out whether the email contains relevant information or not (Burgess et al., 2005), creating high email load. Furthermore, Hacker (2021) argued that even emails that are obviously relevant often contain a lot of information that is not significant for immediate action regulation on momentary executed work tasks and therefore create unnecessary effort in reading and processing (Addas and Pinsonneault, 2018). Following the proposition that communication-related emails hinder effective action regulation, they are likely causing a regulation obstacle that becomes evident in high email load perceptions.

In contrast, task-related emails contain work-tasks, task assignments, task results, or particular questions on work items and are therefore likely to be addressed to a specific recipient (Addas and Pinsonneault, 2018). Being connected to primary goals, task-related emails should be an inherent part of one’s work that certainly requires regulatory effort but is less likely to impede action regulation toward prioritized goals. This assumption is supported by Addas and Pinsonneault (2018), who showed that emails containing pertinent information for primary tasks (called congruent emails) were unrelated to workload, whereas emails containing irrelevant information (i.e., incongruent emails) were positively related to it. Thus, task-related emails are more likely to convey information of higher quality compared to communication-related emails, thereby rendering task-related emails less detrimental to effective action regulation. For example, a study by Piecha and Hacker (2020) revealed that the effect of receiving new tasks on information overload was weaker (*r* = 0.32) than the effect of information quantity (*r* = 0.52).

Furthermore, compared to face-to-face conversations, email is a more informal communication medium that often contains ambiguous and superficial information (Brown et al., 2014; Nantz and Drexel, 1995), a typical aspect of regulation uncertainty. This shortcoming of email, however, applies more often to communication-related emails rather than task-related emails, as the former are typically sent to larger recipient groups and contain more general information. In contrast, task-related emails are tailored to the respective recipient, as the sender of a task-related email often has a personal interest in articulating information clearly, aiming to enhance work outcomes.

Considering the aforementioned differentiation between received and processed emails, we expect that the volume of received emails should hinder effective action regulation due to their nature as regulation obstacles or regulation uncertainties. Conversely, while the quantity of processed emails may impose significant demands on action regulation, its impact is expected to be milder. In conjunction with the distinction between communication- and task-related emails, our theorizing suggests focusing on the volume of received communication- and task-related emails when predicting email load. Nevertheless, we control for the number of processed communication- and task-related emails to investigate the value of this differentiation. Accordingly, we expect:

*Hypothesis 5*: The number of communication-related emails received has a stronger positive relationship with email load than the number of task-related emails received.

## 4 The present studies

Both studies were conducted prior to COVID-19. In the two-wave Study 1, we examined the competing hypotheses of whether high email load uniquely predicts strain, well-being, and work-related stressors, or whether it arises as a consequence of these variables. Study 2 broadens the perspective on high email load by examining email-specific antecedents, making distinctions between received and processed, as well as between communication- and task related email volume. Figure 2 illustrates our research model and the respective contributions of each study to enhance our understanding of email load.

## 5 Study 1 method

### 5.1 Sample and procedure

Participants were recruited through a German panel management and online research company adhering to the International Standardization Organization (ISO) 20252:2019 standards. These standards specify the operational criteria for panel providers and outline methods for assessing their quality. All individuals were instructed that participation was voluntary and that they were allowed to withdraw at any time. To ensure a high response rate, each participant received a compensation of 35 Euro after completion of the study, which also included a diary study that comprised 10 sessions between the two waves. However, this diary study was unrelated to our focal research question and was instead reported in another publication (for data transparency, see Appendix in Supplementary material), so it is not considered further in this article.

The time lag between the two measurement points was set at 2 weeks, considering the following aspects: first, the outcome variables of our study (emotional irritation as strain reaction and affective well-being) can be classified as mid-term stress reactions (Dormann and van de Ven, 2014), which should fluctuate in shorter periods than variables such as depressive symptoms or somatic complaints. Second, Dormann and Griffin (2015) called for the use of shorter time lags than typically used for methodological reasons. They argued that cause-effect relationships are often small and therefore “likely to be obscured” (p. 499) if the time lags are too large. Third, the effects proposed in the hypotheses are based on an exposure-time model (Dormann and van de Ven, 2014; Frese and Zapf, 1988), i.e., adverse effects on strain and well-being are assumed to increase or decrease with increasing or decreasing email load. It has been recommended to use shorter time lags for this model to avoid interim effects such as seasonal fluctuations or disturbances, which bias the cross-lagged effects downward (Dormann and van de Ven, 2014). Thus, 2 weeks seemed appropriate for hypothesis testing.

In total, 444 employees provided complete responses to the two surveys. On average, participants were 43.20 years old (*SD* = 10.94) and half of them (49.8%) were female. As indicated by the average weekly working hours (*M* = 36.00, *SD* = 7.56), our sample included mostly full-time employees. Mean job tenure was 14.33 years (*SD* = 10.94). Most participants held a white-collar position (e.g., administrative clerks, education and academia jobs, sales and customer service jobs, jobs in finance and accounting) without leadership responsibilities (45.5%), followed by 26.1% participants with leadership responsibilities, 18.9% blue collar workers (e.g., transportation and logistics jobs, craftspeople), and 4.5% civil servants. Eight participants were self-employed (1.9%), the remaining 14 participants were either apprentices or did not provide any information about their profession. Nearly half of the sample (*n* = 196; 44.1%) worked from home or remotely for at least part of the time, with the majority consisting of leaders or white-collar employees. Only 10 participants reported working from home more than 50% of the workweek, while 27 participants worked remotely for more than 50% of their workweek. Our sample consisted of fairly-well educated individuals, with 22.3% having a university degree and 19.1% having a high school graduation (A-levels).

### 5.2 Measures

#### 5.2.1 Email load

Email load was assessed using the four email-related items from the Cyber-Based Information Overload Scale by Misra and Stokols (2012). Response options ranged from 0 = *never* to 4 = *very often*. Examples were “How often have you forgotten to respond to important email messages?” and “How often have you felt that you receive more email attachments than you can handle?” Internal consistency measured with McDonald’s *ω* (McDonald, 1999) was 0.86 at T1 and 0.89 at T2.

#### 5.2.2 Time pressure

Time pressure was measured with the five-item scale from the Instrument for Stress-Oriented Task Analysis (ISTA; Irmer et al., 2019; Semmer et al., 1995). Items required responses on a 5-point scale that ranged from 1 = *rarely/never* to 5 = *very frequently (multiple times an hour)*. One example was: “How often do you work under time pressure?.” Omega indicated good scale reliability, both at T1 (ω = 0.87) and T2 (ω = 0.87).

#### 5.2.3 Work interruptions

To measure work interruptions, the 5-item scale from the ISTA was employed (Irmer et al., 2019; Semmer et al., 1995). Respondents rated items such as “Do you often have to interrupt your work because something important comes up?” on a 5-point scale ranging from 1 = very *rarely/never* to 5 = *very often, several times an hour*. The omega coefficient of ω = 0.79 at both T1 and T2 indicated good reliability of the scale.

#### 5.2.4 Strain: irritation

We took the emotional irritation subscale from the irritation scale by Mohr et al. (2005; see also Mohr et al., 2006) as an indicator of psychological strain. The five items required responses on a 7-point scale that ranged from 1 = *does not apply at all* to 7 = *fully applies*. An example was: “When I came home tired after work, I felt rather irritable.” Omega coefficient indicated good reliability at both T1 (ω = 0.90) and T2 (ω = 0.91).

#### 5.2.5 Affective well-being

Employees’ affective well-being was assessed using five items from the WHO-5 well-being index (Bech et al., 2003). Participants rated their well-being in the last 2 weeks on a 6-point scale ranging from 0 = *never* to 5 = *all the time*. A sample item was “In the last 2 weeks, I felt calm and relaxed.” McDonald’s ω was 0.91 at both measurement times.

### 5.3 Statistical analysis

Means, standard deviations, internal consistencies, and intercorrelations are shown in Table 1. Using structural equation modeling (SEM), data were analyzed in Mplus 8.8 (Muthén and Muthén, 1998–2017). All constructs were modeled as latent variables based on item parcels. Item parcels help in reducing the likelihood of correlated residuals, a common issue when numerous indicators are used (Little et al., 2013). Additionally, item parceling reduces the number of estimated parameters, thereby mitigating bias in estimates (Bandalos, 2002). The item parcels were created in line with the recommendations by Little et al. (2013). The allocation of items to parcels was the same for T1 and T2. To assess whether the parceling technique yielded significantly different estimates for the regression paths, we also analyzed a non-parceled model. The differences in results between these models are reported in the results section. Our data had hardly any missing responses (<5%) so that full-information maximum likelihood estimation was employed, as proposed by Raykov (2005). We considered the percentage of time spent working from home and working remotely as potential control variables, given that working away from the office has been associated with an increase in virtual communication (e.g., Singh et al., 2022). However, as shown in Table 1, there were hardly any significant correlations between these variables and the study variables. Furthermore, including these controls in the model did not alter the regression coefficients, so the results are reported without them.

**Table 1:** Means, standard deviations, reliabilities, and intercorrelations among the Study 1 variables.

		*M*	*SD*	ω	1	2	3	4	5	6	7	8	9	10	11	12	13
1	Age	43.21	11.87	–	–												
2	Sex (1 = female; 2 = male)	1.50	0.50	–	−0.03	–											
3	% working from home	5.21	15.21	–	0.09	−0.01	–										
4	% working remotely	9.20	19.40	–	0.11*	−0.15**	0.07	–									
5	Email load T1	1.29	0.91	0.86	−0.08	0.01	0.13***	<0.01	–								
6	Email load T2	1.39	0.97	0.89	−0.06	−0.01	0.08	−0.01	0.74***	–							
7	Time pressure T1	3.00	0.95	0.87	−0.04	0.06	0.02	0.04	0.36***	0.30***	–						
8	Time pressure T2	3.08	0.92	0.87	−0.03	0.08	0.01	−0.01	0.37***	0.44***	0.75***	–					
9	Work interruptions T1	3.09	0.89	0.79	−0.16***	0.10*	−0.07	−0.14**	0.36***	0.28***	0.54***	0.46***	–				
10	Work interruptions T2	3.16	0.84	0.79	−0.17***	−0.08	−0.05	−0.11*	0.37***	0.43***	0.51***	0.59***	0.72***	–			
11	Irritation T1	2.59	1.25	0.90	−0.20***	0.04	0.01	0.01	0.25***	0.18***	0.31***	0.21***	0.18***	0.14**	–		
12	Irritation T2	2.79	1.34	0.91	−0.20***	0.02	0.02	−0.03	0.25***	0.29***	0.27***	0.30***	0.17***	0.17***	0.69***	–	
13	Affective well-being T1	4.00	1.03	0.91	0.18***	−0.04	0.02	0.04	−0.09*	−0.11*	−0.11*	−0.11*	−0.04	−0.04	−0.47***	−0.49***	–
14	Affective well-being T2	3.91	1.01	0.91	0.19***	−0.13**	< 0.01	0.10*	−0.15**	−0.13**	−0.20***	−0.18***	−0.18***	−0.13**	−0.42***	−0.52***	0.66***

Omega coefficients (ω) are provided to evaluate scale reliabilities.

*p < 0.05, **p < 0.01, ***p < 0.001 (two-tailed).

An important prerequisite for valid estimation of lagged effects is strict metric invariance (Finkel, 1995). Thus, we constrained all factor loadings, parcel intercepts, and residual variances of the parcels to be equal at both measurement times. In addition, we assumed tau equivalence for the measurements with item parcels, except for email load. For email load, the chi-square statistic indicated that tau equivalence did not apply, so these restrictions were omitted. Consistent with recommendations in the literature, we allowed the corresponding error terms of the item parcels over time to covary (Finkel, 1995; Little et al., 2007). Imposing these invariance constraints resulted in a good model fit and was used for hypothesis testing.

Model fit was assessed with the chi-square goodness-of-fit test, the Root Mean Square Error of Approximation (RMSEA), and the Comparative Fit Index (CFI). Following Schermelleh-Engel et al. (2003), chi-square values smaller than the twofold degrees of freedom, RMSEA values smaller than 0.05 and CFI values greater than 0.97 point to a good model fit. The full model presented in Figure 1, with further details on standard errors and autoregressive effects provided in Table 2, fit the data well, as indicated by χ^2^ = 249.55, *df* = 143, χ^2^/*df* = 1.75, RMSEA = 0.041, and CFI = 0.985.

**Table 2:** Autoregressive and lagged effects in Study 1.

	Email load T2		Time pressure T2		Work interruptions T2		Irritation T2		Affective well-being T2	
	β	*SE*	β	*SE*	β	*SE*	β	*SE*	β	*SE*
Email load T1	0.82***	0.03	0.11**	0.04	0.13**	0.05	0.08*	0.04	−0.01	0.05
Time pressure T1	0.06	0.06	0.78***	0.05	0.21***	0.06	0.03	0.06	−0.02	0.06
Work interruptions T1	−0.05	0.05	0.01	0.05	0.60***	0.05	0.02	0.06	−0.14*	0.06
Irritation T1	−0.08*	0.05	−0.11*	0.05	−0.09*	0.05	0.56***	0.05	−0.09*	0.05
Affective well-being T1	−0.08*	0.05	−0.07*	0.04	−0.02	0.05	−0.24***	0.05	0.64***	0.04

Standardized regression coefficients displayed.

*p < 0.05, **p < 0.01, ***p < 0.001 (one-tailed).

## 6 Study 1 results

In Hypothesis 1a, we predicted that high email load has a positive lagged effect on strain, even when controlling for time pressure and work interruptions. The results shown in Table 2 revealed a positive lagged effect (*β* = 0.08, *p* = 0.044) on emotional irritation in line with expectations. However, Hypothesis 1b concerning the lagged effect on affective well-being was not supported, as the lagged effect was close to zero and insignificant (β = −0.01, *p* = 0.414).

Hypothesis 2 addressed reversed causal relationships from strain and well-being on email load. Contrary to expectations, the lagged effect from strain was negative and significant, indicating that strained employees reported less email load 2 weeks later (β = −0.08, *p* = 0.045). For well-being, however, the result was in line with our prediction: The higher well-being was at T1, the lower was email load at T2 (β = −0.08, *p* = 0.039). Thus, Hypothesis 2 found partial support. In the supplemental analyses, no reverse causal relationships were significant (see Supplementary Table S1).

**Figure 3:**
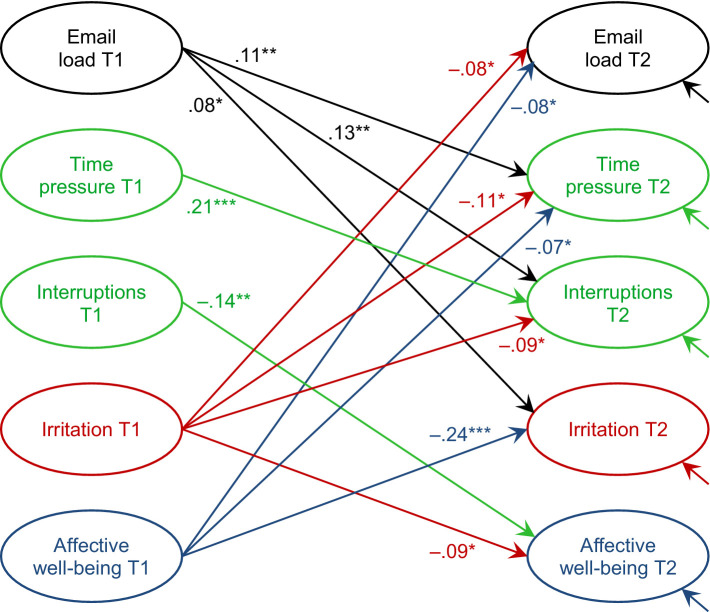
Cross-lagged effects in Study 1. Standardized regression coefficients displayed. For the sake of clarity, autoregressive effects and cross-sectional (residual) correlations are not displayed. On the left side, the significant cross-lagged coefficients from email load (black) and the other two stressors are depicted (green). On the right side, the significant cross-lagged effects from irritation and well-being are depicted (red and blue). *p < 0.05, **p < 0.01, ***p < 0.001 (one-tailed).

In Hypothesis 3, we predicted that time pressure and work interruptions lead to high email load over time. However, as shown in Table 2, the lagged effects were not significant for either time pressure or interruptions, so Hypothesis 3 was not supported. In contrast, the results supported the competing Hypothesis 4: High email load had a positive lagged effect on both time pressure (β = 0.11, *p* = 0.003) and work interruptions at T2 (β = 0.13, *p* = 0.003), supporting the notion that email load increases stressors over time. An overview of the model results, including all significant lagged effects, is shown in Figure 3.

To check whether the use of item parceling introduced bias in our results, we also analyzed a non-parceled model. The initial non-parceled model, with all invariance constraints in place, showed a poorer fit, indicated by χ^2^ = 2041.99, *df* = 1,072, χ^2^/*df* = 1.90, RMSEA = 0.045, and CFI = 0.936. We subsequently improved the model fit by releasing the residual covariances and invariance constraints that had the most significant impact on model inflation, as indicated by modification indices greater than 20. The adjusted model achieved an acceptable fit, with χ^2^ = 1885.09, *df* = 1,057, χ^2^/*df* = 1.78, RMSEA = 0.042, and CFI = 0.945, though it still did not perform as well as the parceled model. Notably, the measurement models for irritation and well-being remained suboptimal, showing numerous residual covariances with other items. Therefore, we opted to retain the parceled model to adhere to stringent criteria for optimal modeling. Although the regression coefficients showed only minimal differences (Δβ < 0.04), two discrepancies were observed concerning Hypothesis 2 (see Supplementary Table). Specifically, the non-parceled model showed no significant lagged effect from irritation on email load and only a marginally significant effect from well-being on email load.

## 7 Study 1 discussion

High email load has long been considered a source of stress, but due to the cross-sectional nature of most studies to date, there is little empirical evidence to support a causal relation. Our findings based on a two-wave design are in line with previous research that document a positive relationship with employee strain (Brown et al., 2014; Reinecke et al., 2017) and confirm, for example, the longitudinal study by Misra and Stokols (2012), who identified a positive lagged effect of “cyber-based” load on strain in a sample of college students. Together with quasi-experimental field research (e.g., Mark et al., 2012) and intervention studies (e.g., Soucek and Moser, 2010), our findings underscore that high email load may actually be a cause of strain, thus confirming the view that it is a regulation problem according to action regulation theory (Hacker, 2003).

With respect to well-being, the data did not support a lagged effect of email load. The reason for the non-existent effect could be that well-being, in contrast to strain, is considered as a more distal outcome (Dormann and van de Ven, 2014; Dormann and Zapf, 2002) and is therefore likely to be affected over a longer period than two-weeks. This explanation is consistent with the notion that negative feelings tend to accumulate over time and thus impair employee well-being in the long term (Reinke and Chamorro-Premuzic, 2014). An alternative explanation might be that strained individuals are more likely to disengage from their work tasks (see Taris et al., 2005; van Dierendonck et al., 2001), leading to a lower reported email load.

Our results provided also support for reverse causation, but with an opposite finding for strain than expected: Based on the stressor perception hypothesis (de Lange et al., 2004; Guthier et al., 2020), we expected that strained employees will experience more email load, but indeed they reported less. A possible explanation could be that emotional irritation refers to immediate consequences of stress (Dormann and van de Ven, 2014; Dormann and Zapf, 2002). We know from occupational stress research that employees are likely to activate compensatory effort when stressed (Meijman and Mulder, 1998), so the employees who reported higher levels in irritation at T1 were likely those who had already activated additional resources to cope with high email load as their sources of stress. Because of compensatory regulation, stressed employees may have been at least partially successful in managing the email flood in the meantime. However, as pointed out by Hockey (1997), maintaining effort under stress becomes increasingly aversive, which may explain why well-being, as a more distal outcome (Dormann and van de Ven, 2014), had a negative lagged effect on email load, as expected. Alternatively, strained individuals might already have disengaged from work, which is why they report less email load later on, corresponding to research on employee burnout (Taris et al., 2005; van Dierendonck et al., 2001).^1^ This explanation is further consistent with the finding that irritation is also linked to lower levels of time pressure and interruptions (see Table 2).

Lastly, our study presents evidence supporting the notion that high email load contributes to elevated work stressors, with no substantiation for reverse causation. In contrast to previous studies (e.g., MacDonald et al., 2011), this finding implies that email load is more likely a cause than a consequence of work stressors. Moreover, it indicates that email load cannot be attributed to the co-occurrence of other work stressors but is rather conceptually distinct, answering the question about construct relevance raised by Hu et al. (2021). However, since no work-related antecedents for email load were identified in Study 1, it remains unclear which aspects of one’s work contribute to it. Based on the findings of this study and the suggestions by Wang et al. (2020), a closer examination of the nature of the emails themselves appears promising, which will be addressed in Study 2.

## 8 Study 2 method

### 8.1 Sample and procedure

Participants were recruited via social and professional networks as well as via advertisement in a German popular science magazine and an online trading platform. Each participant was instructed about the purpose of the study and anonymity was assured. After informed consent was obtained, participants answered the questionnaire. A total of 315 participants completed the study, 55 of which had to be excluded from further consideration because they did not belong to the working population (e.g., students and interns). Three additional participants were excluded due to reporting implausible values on both the number of emails read and processed (≥150 mails per day) and the time spent on emails (≥8 h per day). Mean age in the final sample of 257 participants was 30.98 years (*SD* = 9.51), 66.9% of whom were female. One person did not provide gender information. Participants exhibited a high level of educational attainment, with 31.6% holding a university degree and 47.1% holding a high school diploma (A-levels). On average, participants reported working 34.38 h per week (*SD* = 9.03). A minority of participants (less than 20%, *n* = 50) held a leadership position. The sample primarily consisted of white-collar workers (84.9%) from diverse industries, including financial services, education, healthcare, and customer service. A smaller portion of participants were blue-collar workers, such as those in construction (4.7%), or worked as civil servants in public administration (3.1%). A total of 108 participants reported working from home or remotely; however, only 16 of them worked offsite for more than 50% of their time.

### 8.2 Measures

Participants were instructed to open their email client to check the number of emails related to each function when responding to the questions below. The two functions were described in detail so that participants were able to differentiate. More precisely, communication-related emails were described as “used to coordinate with colleagues or to exchange information” and as not containing any work tasks. Task-related emails were described as “sending work task instructions via email or transmitting their solutions.”

#### 8.2.1 Communication-related emails received and processed

Using the two items measuring the number of received emails from Brown et al. (2014), participants were asked to indicate how many emails in their inbox relate to communication or information purposes. One example is: “How many emails do you receive per day related to the above purpose?.” The other four items taken from Brown et al.’s (2014) email quantity scale were used to assess the number of emails processed during a workday. These were: “How many emails do you read per day related to the above purpose?,” “How many emails do you send per day related to the above purpose?,” “Please tell us how many emails you read with the above-mentioned purpose during the last full working day.,” and “Please tell us how many emails you sent with the above-mentioned purpose during the last full working day.” McDonald’s *ω* pointed to good scale reliability, both for communication-related emails received (ω = 0.86) and processed (ω = 0.73; Table 3).

**Table 3:** Means, standard deviations, reliabilities, and intercorrelations among the Study 2 variables.

		*M*	*SD*	ω	1	2	3	4	5	6	7	8	9	10
1	Age	30.98	9.49	–	–									
2	Sex (1 = female; 2 = male)	1.34	0.48	–	0.18**	–								
3	% working from home	5.62	14.87	–	−0.03	0.01	–							
4	% working remotely	8.38	16.67	–	0.13*	0.15*	0.16*	–						
5	Leadership function (1 = no; 2 = yes)	1.20	0.40	–	0.21***	0.21***	0.12	0.14*	–					
6	Time pressure	2.97	0.87	0.86	−0.01	−0.07	0.01	0.01	0.26***	–				
7	Communication-related emails received	10.15	14.18	0.86	−0.03	> −0.01	0.03	0.07	0.28***	0.27***	–			
8	Communication-related emails processed	5.60	9.91	0.73	0.02	−0.10	0.04	0.03	0.26***	0.24***	0.76***	–		
9	Task-related mails received	6.90	11.43	0.92	−0.03	−0.04	−0.05	0.02	0.08	0.22***	0.45***	0.36***	–	
10	Task-related mails processed	4.68	7.72	0.92	−0.04	−0.06	−0.03	0.01	0.12*	0.24***	0.53***	0.58***	0.86***	–
11	Email load	2.47	0.80	0.81	0.08	−0.02	0.10	0.01	0.16**	0.47***	0.38***	0.36***	0.31***	0.37***

Omega coefficients (ω) are provided to evaluate scale reliabilities. Correlations are based on the untransformed values of the email variables.

*p < 0.05, **p < 0.01, ***p < 0.001 (two-tailed).

#### 8.2.2 Task-related emails received and processed

We used the same two items as for communication-related emails to assess the number of emails received pertaining to specific work tasks. Item wording was slightly adjusted to emphasize the task aspect (e.g., “How many emails containing work tasks do you receive per day?”). To measure the number of task-related emails processed, the same four items were used as for communication-related emails. An example was: “How many emails with work tasks do you send per day?.” The coefficient ω for both scales was 0.92, indicating high reliability.

#### 8.2.3 Email load

Email load was assessed with the same scale as in Study 1. Internal consistency with an omega of ω = 0.81 was similarly high as in Study 1.

### 8.3 Statistical analysis

Prior to hypothesis testing, we analyzed the distribution of the number of communication- and task related emails received and processed. The variables exhibited substantial skewness (3.21 < skewness < 5.02), necessitating transformation to the natural logarithm for hypothesis testing (Becker et al., 2019). Hypotheses were tested using multiple linear regression analysis in Mplus 8.8 (Muthén and Muthén, 1998–2017), controlling for time pressure (based on the rationale discussed above) and leadership position. As in Study 1, we considered the proportion by which individuals worked from home or remotely as potential control variables as well. However, as these variables showed no significant correlations with the study variables, they were excluded from further analysis. We report fully standardized results based on maximum likelihood estimation.

## 9 Study 2 results

The number of communication- and task-related emails received were positively but only moderately correlated (*r* = 0.45), indicating that participants were able to distinguish between the two types. The same was true for communication- and task-related emails processed (*r* = 0.45). Moreover, all types of emails showed significant positive bivariate relationships with email load.

In Hypothesis 5, we expected a stronger effect of communication-related emails received compared to task-related emails received: Results showed that only communication-related emails received were positively related to email load (*β* = 0.46, *p* < 0.001). Task-related emails received were unrelated to email load, suggesting a suppressor effect. The difference between these two relationships was also significant, as shown by a z-test in Model 2: *Δ*[β/SE] = 2.58, *p* = 0.005 (one-tailed), supporting Hypothesis 5. A comparison of Model 1 without controls and Model 2 including time pressure and leadership revealed no significant differences (Table 4).

**Table 4:** Communication- and task-related emails predicting email load (Study 2).

	Model 1				Model 2			
			95% CI interval				95% CI interval	
	β	*SE*	LLCI	ULCI	β	*SE*	LLCI	ULCI
Control variables								
Leadership function (1 = no, 2 = yes)					0.01	0.05	−0.10	0.09
Time pressure					0.33***	0.05	0.12	0.32
Study variables								
Communication-related emails received	0.46***	0.14	0.19	0.73	0.37**	0.13	0.14	0.66
Communication-related emails processed	−0.16	0.14	−0.38	0.15	−0.12	0.12	−0.34	0.17
Task-related mails received	0.14	0.14	−0.23	0.33	0.17	0.13	−0.20	0.34
Task-related mails processed	0.20	0.15	−0.03	0.54	0.12	0.14	−0.08	0.48

Standardized regression coefficients displayed. The coefficients are based on log transformed email variables. CI, confidence interval; LLCI, lower-level confidence interval; ULCI, upper-level confidence interval.

*p < 0.05, **p < 0.01, ***p < 0.001 (one-tailed).

## 10 Study 2 discussion

Based on action regulation theory (Frese and Zapf, 1994; Hacker, 2003), we distinguished between received and processed emails and between communication-related and task-related emails to better understand the causes of high email load (see Wang et al., 2020). Results confirmed that communication-related emails received are responsible for high email load and thus can be considered the most detrimental aspect of email use. Contrary to expectations, task-related emails received were not related to email load, probably because they contain less irrelevant and unclear information, but rather provide some benefit for action regulation related to primary tasks. This explanation coincides with the results of Rosen et al.’s (2019) study, in which goal progress was not affected by high email demands when email was a central part of the job.

Moreover, neither the number of processed communication-related nor the number of processed task-related emails were related to email load. A reason for this finding might be that processing emails can be scheduled for convenient times (“batching emails”; Dabbish and Kraut, 2006) and thus cannot be considered as a regulation obstacle. It is important to note that the correlative results differed considerably from the results of multiple regression analysis: Whereas all types of emails were positively related to email load (Table 1), only communication-related emails received were positively related to email load when all four types were considered simultaneously. This pattern suggests a suppressor effect, indicating that task-related emails received and all processed emails may have a dual impact. On one hand, they demand cognitive and emotional effort, contributing to increased email load. On the other hand, these emails are directly aligned with work tasks and can lead to feelings of accomplishment and success. When controlling for the most negative email class—communication-related emails received—the negative impact of task-related and all processed emails on email load disappears, which is a common finding for challenge stressors (e.g., Widmer et al., 2012). Thus, previous results may have been subject to significant bias due to the omission of considering the distinct effects associated with whether individuals solely receive or actively process emails, as well as the specific function of email (e.g., Brown et al., 2014). Findings on email load that have been based on the email quantity scale (e.g., Dabbish and Kraut, 2006) or the email demands scale (Steffensen et al., 2022), for example, may therefore have been falsely attributed to email use in general, whereas our findings suggest a finer distinction of email classes. This may stimulate future research to examine the potential dual process in more detail.

## 11 General discussion

The main goal of the present work was to advance our knowledge of high email load by examining its effects on strain and well-being in the context of coexisting stressors, and by distinguishing between certain classes of email that are responsible for experiencing email load. Overall, our findings indicate that email load uniquely predicts strain and increases work stressors over time, as indicated by positive lagged effects on irritation, time pressure and work interruptions. There were no reversed effects of time pressure and work interruptions on email load, suggesting that it is not the consequence of too much work stress. The results of Study 2 showed that one particular class of email is associated with high email load, namely the volume of communication-related emails received. In contrast, task-related emails and both classes of processed emails were not related to email load.

Two important findings warrant further discussion. First, our results suggest that email load is a regulation problem that predicts employee strain over and above co-existing stressors. This finding is consistent with previous studies (e.g., Misra and Stokols, 2012; Soucek and Moser, 2010) and a recent meta-analysis on information overload (Graf and Antoni, 2023), but at the same time adds to the literature by showing that high email load has an effect that goes beyond previously known stressors (time pressure, interruptions). As time pressure is commonly considered as an overtaxing regulation and work interruption as a regulation obstacle (Frese and Zapf, 1994), the significant results despite controlling for these stressors could be an indication that email load represents a regulation uncertainty. However, this interpretation is relatively speculative and needs to be further substantiated in future studies that explicitly examine the cognitive processes underlying all three forms of regulation problems related to email use. In addition, our results indicate that high email load hampers one’s work even further, given the lagged effects on time pressure and interruptions. This can be explained by the time employees need to deal with their emails during their workday (Barley et al., 2011; Mark et al., 2016) and the time it takes employees to resume work when emails barge in (with signals), both of which reduce the time available to complete scheduled tasks.

Second, in line the propositions of action regulation theory (Frese and Zapf, 1994; Zacher and Frese, 2018), we found that the main source of email load is the number of communication-related emails received. Again, this may suggest that email load is primarily a regulation uncertainty, as communication-related emails are mostly unrelated to salient work tasks and often reach recipients without cause, so it is unclear how to deal with them. The prominent role of communication-related emails identified in our study contrasts with research that attributes the cause of high email load to the number of unfinished tasks in the emails received (Bellotti et al., 2005; Rennecker and Derks, 2012), as communication-related emails do not involve such. Thus, the distinction between task- and communication-related emails based on previous research (Addas and Pinsonneault, 2018; Rice and Leonardi, 2014; Wang et al., 2020) brings a new perspective on email use, suggesting that emails containing less relevant information are more harmful. Together with the study by Dietz et al. (2022) on information and communication technology use more generally, our work represents an initial test of differentiating email function to offer more accurate insights into the origins of email load.

In sum, our studies clearly suggest that email demands should no longer be measured as a higher-order construct subsuming both emails received and processed and both email functions. Studies that have employed the scales by Brown et al. (2014) or Dabbish and Kraut (2006) may have underestimated the negative consequences of certain types of emails, and have falsely attributed negative effects to other classes of email. Our results for task-related emails and for both classes of processed emails emphasize the functional role that email can have at work. These emails might even lead to higher work engagement when email is a central tool to accomplish tasks (Rosen et al., 2019). However, by assessing the specific classes of emails instead of overall quantity or quality of email, the positive effects of certain emails classes need to be investigated in future work before such conclusions can be reliably drawn. In this context, it seems valuable to explore the role of additional communication media, such as in person meetings, video conferencing, or project management software to better understand when work-related communication leads to high information load.

## 12 Limitations and future research

A few limitations should be considered when interpreting the results of our two studies. First, both studies relied on self-reports, which are subject to common-method variance and may have inflated the relationships (Podsakoff et al., 2024). However, as both methodological approaches (i.e., longitudinal and cross-sectional) produced comparable and integrative results but were not affected by this bias to the same extent, our findings are unlikely to be the result of common method variance alone. By separating measurements over time and controlling for autoregressive effects in Study 1, associations between predictor and outcome variables are less likely to be overestimated (Podsakoff et al., 2024). Another argument against problems with artificially inflated relationships is that in Study 2, participants were instructed to check the number of emails of each type in their email programs, making these measurements more objective and valid (see also Conway and Lance, 2010).

Second, we refrain from drawing causal conclusions from our studies because omitted variables, interaction terms, or polynomial terms could have accounted for the effects identified in our study (Antonakis et al., 2010), which cannot be explicitly tested with the data available. However, the analysis of cross-lagged effects in Study 1 can provide valuable insights into the direction of effects. Moreover, reverse effects in Study 2 (email load influencing the number of emails) are unlikely because participants were asked not to report them from memory but to count them directly in their email app. We therefore believe that our results can validly contribute to a better understanding of high email load. Nevertheless, future longitudinal research should include a third or even more waves and could apply growth curve modeling to determine which predictors increase or decrease email load over time. To understand temporal and short-term dynamics, it also seems promising to conduct diary studies, ideally with multiple measurements per day. This may allow to examine changes in well-being as a direct function of task- or communication-related emails received and processed.

Third, the sample size of Study 2 might represent a limitation, particularly considering the substantial intercorrelations among email predictors (see Table 3), which contributed to relatively large standard errors in the regression analysis. We conducted a Monte Carlo simulation to determine the power with which a medium-sized regression effect of *β* = 0.30 could be detected. The simulation, based on the obtained means, variances, and intercorrelations, indicated that in 98% of 500 random replications, the relationship between communication-related emails received and email load would have been detected with 80% power. Consequently, while the results appear sufficiently credible, replication in future studies is necessary to confirm these findings.

Nonetheless, these limitations result in concrete next steps for future research. Given the role interindividual characteristics play for the experience of email load (e.g., Reinke and Chamorro-Premuzic, 2014; Russell et al., 2022), prospective studies could explore how the different classes of email affect employees depending on their personality. There is reason to believe that communication-related emails are more likely detrimental for introverted individuals, whereas extraverted may also satisfy their needs for relatedness and affiliation and therefore react less negatively (Russell et al., 2022). In a similar vein, effects of email on well-being may depend on how employees manage their inbox. Extant literature suggests a moderating role of email-handling strategies (Gupta et al., 2006, 2011; Russell et al., 2007), however, we do not know which strategy is helpful for which class of email. We recommend the use of longitudinal and experience sampling field studies due to their higher ecological validity. Additionally, incorporating alternative communication media or the use of generative artificial intelligence could be a valuable tool for managing high email load (Stray et al., 2019; Trenerry et al., 2021).

Another next step for future endeavors concerns the construct validity of email load. In our study, email load relied on the definition as a *subjective* feeling of having to read and process more emails than one believes one can actually handle (Dabbish and Kraut, 2006, p. 431), emphasizing the high volume and low quality of emails (Brown et al., 2014; Gupta et al., 2011; Sobotta, 2016; Stich et al., 2019b). However, there are also scholars who suggest that email load is a state of overwhelming the user, thus stressing the individual response (Graf and Antoni, 2023; Sumecki et al., 2011). As a result, there seems to be a conceptual overlap between a stressor and a strain definition in high email load that needs to be further clarified.

## 13 Implications for theory and practice

This article makes several meaningful contributions: First, recourse to concepts from action regulation theory (Frese and Zapf, 1994; Hacker, 2003) has proven helpful in understanding the nature of high email load. Our theoretical considerations built on the study by Russell et al. (2007) and Addas and Pinsonneault (2018), who used the tenets of action regulation theory to explain how people deal with received and sent emails and showed that a clear theorization can help uncover issues of email use that have been insufficiently illuminated. In a similar vein, the three regulation problems defined in action regulation theory allowed us to differentiate between different classes of emails based on how they interfere with goal attainment, going beyond previous research that grouped all classes of email demands into a single construct (Brown et al., 2014; Dabbish and Kraut, 2006).

Second, given that email load preceded both strain as well as time pressure and work interruptions, our findings complement research considering email demands from an occupational health perspective, as was done by Steffensen et al. (2022) or Kubo et al. (2021). High email load negatively impacts employees, but our results suggest that this perception results primarily from communication-related emails, which are often disconnected from actual work and unrelated to goals. Certainly, a single email is not disruptive enough to increase strain, but the sum of these mails creates a high load and exhausts employees. Thus, communication-related emails can be considered as daily hassles (Kanner et al., 1981) or a hindrance stressor within the challenge-hindrance stressor framework (Cavanaugh et al., 2000; LePine et al., 2005) that should be reduced to a minimum.

Third, another conclusion relates to the interaction type and function of emails that were not associated with email load. We argued that the number of task-related emails received as well as all processed emails, although they make work more difficult when their number increases, contain a functional component (Addas and Pinsonneault, 2018). This led us to believe that although they represent an overtaxing regulation, they are related to email load to a much lesser extent than incoming communication-related emails. However, our data showed that task-related emails received, and both classes of processed emails were not uniquely related to email load. This is consistent with findings on other overtaxing regulations such as time pressure or concentration demands (Kern and Zapf, 2021; Widmer et al., 2012), which have been explained using the tenets of the challenge-hindrance stressor framework (Cavanaugh et al., 2000). It could be that receiving task-related emails as well as the requirement to process emails are challenge demands that include both a negative and a positive component, resulting in a net null effect. This perspective is not explicitly addressed in action regulation theory, which traditionally focuses on the proposition that all regulation problems act as stressors leading to negative outcomes. However, our findings, along with evidence from research on challenge stressors, suggest that the conceptual framework of overtaxing regulations may need to be revised to account more explicitly for potential positive effects. Enhancing action regulation theory with elements from the challenge-hindrance stressor framework could offer a more nuanced understanding of how specific stressors, such as specific email demands, can contribute to motivation and well-being under specific conditions, even while making goal-directed actions more difficult.

In practical terms, it is evident that email will remain the primary communication medium at work (The Radicati Group, 2023), even as the use of alternative media such as instant messaging or project management software continues to grow (Stray et al., 2019; Trenerry et al., 2021). Considering our findings, it is imperative to explore all available avenues to mitigate the burden of high email load. For example, because our study found that incoming communication-related emails contribute to email load, which in turn triggered strain, organizations, particularly supervisors (Rademaker et al., 2023), are well advised to implement policies aimed at minimizing the frequency of communication-related emails. Instead, they should consider alternative ways of distributing information that has no immediate task-relevance, such as using internal websites, information/notice boards, or conducting regular face-to-face meetings. This approach is also beneficial because employees often overestimate how quickly they are expected to respond (Giurge and Bohns, 2021). Alternatively, the increasing reliance on alternative media for task-relevant information, such as Slack, Trello, and Microsoft Teams (Stray et al., 2019; Trenerry et al., 2021), might reduce the need for constant email monitoring. As a result, employees would be less compelled to pay attention to communication-related emails, which could reduce their email load and ensuing detrimental effects.

Another take-home message is directed at the individual: Considering the predominant role of communication-related emails, it appears sensible to offer recommendations for email management specifically tailored to this category of emails. Strategies such as filing emails, filtering, categorizing, analyzing, and prioritizing emails, or limiting email retrieval to certain times of the day have been identified as useful in reducing email load and the resulting strain (e.g., Kushlev and Dunn, 2015; Russell et al., 2007). However, effectiveness of these strategies has not been clearly demonstrated, but this may be because past studies did not distinguish between different classes of emails. For example, filing emails into folders reduced email load in a study by Whittaker et al. (2011) but increased it in a study by Dabbish and Kraut (2006). Storing emails might therefore only be useful if the high load is due to emails that are not primarily related to the tasks at hand. It might be a less effective strategy when high email load comes from the amount of work, or the number of tasks submitted via email. When high email load is a regulation obstacle due to many incoming emails at inconvenient times, other strategies might be effective (e.g., turning automatic alerts off) than when email load is attributed to overtaxing regulation due to many tasks contained in the emails. However, these considerations require further research.

## Data Availability

The raw data supporting the conclusions of this article will be made available by the authors, without undue reservation.

## Footnotes

^1^We thank the anonymous reviewer for this suggestion.
