# Supplementary material for: Drowning in emails: investigating email classes and work stressors as antecedents of high email load and implications for well-being
Source: Front Psychol. 2024 Oct 3;15:1439070. doi: 10.3389/fpsyg.2024.1439070 (PMC11484023; doi:10.3389/fpsyg.2024.1439070)
Supplement: Supplementary file 1 [file Table_1.DOCX]

**Supplementary Table**

*Autoregressive and Lagged Effects in Study 1 Without Item Parceling*

|  | Email  load T2 | | Time  pressure T2 | | Work  interruptions T2 | | Irritation  T2 | | Affective well-being T2 | |
| --- | --- | --- | --- | --- | --- | --- | --- | --- | --- | --- |
|  | β | *SE* | β | *SE* | β | *SE* | β | *SE* | β | *SE* |
| Email load T1 | .82*** | .03 | 12** | .04 | .15*** | .05 | .07* | .04 | –.03 | .05 |
| Time pressure T1 | .05 | .06 | .78*** | .05 | .14** | .06 | .03 | .06 | –.05 | .06 |
| Work interruptions T1 | –.06 | .06 | > –.01 | .06 | .64*** | .05 | < .01 | .06 | –.10† | .06 |
| Irritation T1 | –.06 | .05 | –.09* | .04 | –.06 | .05 | .58*** | .04 | –.08* | .05 |
| Affective well-being T1 | –.06† | .04 | –.06† | .04 | –.02 | .04 | –.21*** | .04 | .63*** | .04 |
| χ^2^(*df*) | 1885.09*** (1057) | | | | | | | | | |
| χ^2^/*df* | 1.78 | | | | | | | | | |
| RMSEA | .042 | | | | | | | | | |
| CFI | .945 | | | | | | | | | |

*Note.* Standardized regression coefficients displayed.

* *p* < .05. ** *p* < .01. *** *p* < .001 (one-tailed).
