# Supplementary material for: Drowning in emails: investigating email classes and work stressors as antecedents of high email load and implications for well-being
Source: Front Psychol. 2024 Oct 3;15:1439070. doi: 10.3389/fpsyg.2024.1439070 (PMC11484023; doi:10.3389/fpsyg.2024.1439070)
Supplement: Supplementary file 2 [file Table_2.DOCX]

**Appendix**

*Data Transparency Table for Study 1*

| Variables in the complete dataset | MS 1  (published) | MS 2  (current) | Not used |
| --- | --- | --- | --- |
| Positive reflection (manipulated) | X |  |  |
| Negative reflection (manipulated) | X |  |  |
| Intense reflection (manipulated) | X |  |  |
| Length and frequency of reporting on social interactions – daily reports | X |  |  |
| Leader-member-exchange T1 and T2 | X |  |  |
| Team-member-exchange T1 and T2 | X |  |  |
| Job satisfaction T1 and T2 | X |  |  |
| Affective work climate T1 and T2 | X |  |  |
| Organizational commitment T1 and T2 | X |  |  |
| Reaction to the study | X |  |  |
| Email overload T1 and T2 |  | X |  |
| Time pressure T1 and T2 |  | X |  |
| Interruptions T1 and T2 |  | X |  |
| Irritation T1 and T2 | X | X |  |
| Affective well-being T1 and T2 |  | X |  |
| Positive affectivity T1 |  |  | X |
| Negative affectivity T1 |  |  | X |
| Big five personality traits T1 |  |  | X |
| Reflexivity T1 |  |  | X |
| Availability expectations T1 and T2 |  |  | X |
| Demographic data | X | X |  |

*Note.* MS 1 refers to a published article, MS 2 to the present article. Only emotional irritation was used in both studies.

In the previously published article, the authors manipulated the intensity and valence of daily reporting of social interactions at work and analyzed the effects of the experimental conditions on the type of daily reporting, as well as on the resulting changes in work-related perceptions (leader-member-exchange, team-member-exchange, affective climate) and outcomes (job satisfaction, organizational commitment).

The current study examines the cross-lagged effects of email overload on emotional irritation and affective well-being without relying on the diary data or the experimental design. Importantly, the experimental factors (intensity or valence of daily reporting) did not affect the focal variables in this manuscript (all *p* > .10).

As part of a possible future manuscript for scale validation, additional variables were collected that were not used in the previous publication or the present manuscript.
